# Impact of cage position on biomechanical performance of stand-alone lateral lumbar interbody fusion: a finite element analysis

**DOI:** 10.1186/s12891-022-05873-x

**Published:** 2022-10-18

**Authors:** Chong Nan, Zhanbei Ma, Yuxiu Liu, Liang Ma, Jiaqi Li, Wei Zhang

**Affiliations:** 1 Department of Spinal Surgery, The Third Hospital of Hebei Medical University, 050000 Shijiazhuang, Hebei Province China; 2 Department of Orthopedic, Central Hospital, Baoding No. 1, 071000 Baoding, Hebei Province China

**Keywords:** Stand-alone lateral lumbar interbody fusion, Cage position, Finite element analysis, Adjacent segment degeneration, Cage subsidence

## Abstract

**Background:**

This study aimed to compare the biomechanical performance of various cage positions in stand-alone lateral lumbar interbody fusion(SA LLIF).

**Methods:**

An intact finite element model of the L3-L5 was reconstructed. The model was verified and analyzed. Through changing the position of the cage, SA LLIF was established in four directions: anterior placement(AP), middle placement(MP), posterior placement(PP), oblique placement(OP). A 400 N vertical axial pre-load was imposed on the superior surface of L3 and a 10 N/m moment was applied on the L3 superior surface along the radial direction to simulate movements of flexion, extension, lateral bending, and axial rotation. Various biomechanical parameters were evaluated for intact and implanted models in all loading conditions, including the range of motion (ROM) and maximum stress.

**Results:**

In the SA LLIF models, the ROM of L4-5 was reduced by 84.21–89.03% in flexion, 72.64–82.26% in extension, 92.5-95.85% in right and left lateral bending, and 87.22–92.77% in right and left axial rotation, respectively. Meanwhile, ROM of L3-4 was mildly increased by an average of 9.6% in all motion directions. Almost all stress peaks were increased after SA LLIF, including adjacent disc, facet joints, and endplates. MP had lower stress peaks of cage and endplates in most motion modes. In terms of the stress on facet joints and disc of the cephalad segment, MP had the smallest increment.

**Conclusion:**

In our study, SA LLIF risked accelerating the adjacent segment degeneration. The cage position had an influence on the distribution of endplate stress and the magnitude of facet joint stress. Compared with other positions, MP had the slightest effect on the stress in the adjacent facet joints. Meanwhile, MP seems to play an important role in reducing the risk of cage subsidence.

## Background

In light of the popularity of minimally invasive Intervertebral fusion surgery, lateral lumbar interbody fusion (LLIF) is increasingly being chosen by surgeons. This technology has been progressively matured and optimized since it was systematically reported in 2006 [1, 2]. LLIF was designed as a retroperitoneal approach to reduce the risk of vascular injury seen with anterior lumbar interbody fusion(ALIF), avoid the disruption of spinal stability seen with transforaminal lumbar interbody fusion (TLIF), and prevent the muscular trauma seen with posterior lumbar interbody fusion (PLIF) [3–5]. Meanwhile, a larger size cage is permitted during LLIF, which can improve the fusion rate [6]. Additionally, this operation has particular advantages in therapy for adjacent segment disease (ASD) and revision surgery, when a conventional posterior decompression has a relatively high dural injury rate, whereas epidural damage during a lateral fusion operation is extremely uncommon [7, 8]. Another minimally invasive superiority of LLIF is that its anatomic approach is away from previous surgical incision and granulation tissue to reduce intraoperative bleeding risk. Louie PK et al. [9–11] demonstrated the availability of the lateral approach procedure for ASD through biomechanical experiments.

The current literature is divided on the rationalization of auxiliary internal fixation. A few experiments [12, 13] have indicated assisted internal fixation exhibits more range of motion (ROM) reduction than stand-alone approach under the same mechanical conditions, especially cage plus bilateral pedicle screw fixation (PSF). Though additional fixation could enhance the mechanical stability to some extent, it also increased the risk of adjacent segment degeneration simultaneously. In practice, since stabilizing structures remain intact, it is suggested that stand-alone lateral lumbar interbody fusion (SA LLIF) can be used for appropriate indications [14]. Marchi et al. [15] found segmental lordosis could be restored by the stand-alone cage. Considering subjective factors such as intraoperative endplate damage, SA LLIF was associated with fusion failure, and higher rate of cage displacement, and high possibility of revision operation due to recurrence of symptoms [16]. To reduce the detrimental impact, placement and size of cage are the uppermost consideration during SA LLIF. Yuan et al. [17, 18] demonstrated that cage size correlated strongly with bone fusion and cage subsidence. And yet in respect to clinical outcomes, the position of interbody fusion cage in LLIF is controversial [19, 20], Qiao et al. [21, 22] reported that disc height and segmental angle could be influenced by cage position. However, little experiment has been designed to investigate the cage position in SA LLIF from a biomechanical point of view.

Accordingly, three-dimensional digital models of lumbar were created to simulate several surgical conditions. The objective of this study was to compare biomechanical performance of various cage positions in SA LLIF by finite element analysis (FEA). The von Mises stress and displacement were used as risk parameters associated with subsidence and adjacent segment degeneration.

## Materials and methods

### Building an intact model

The volunteer we recruited (40-year-old, female, 60Kg, 160 cm) did not have any spine or systemic disease. All procedures were in accordance with the ethical standards of the institutional and national research committee.Written informed consent was acquired before the examination. The normal L3-5 segments of the volunteer were scanned using a computed tomography scanner (Philips, Netherlands, 128 spiral, 120kv, 81mAs, 279mA, Pitch 1. 38). A series of images (Slice Thk 0. 5 mm ) were exported and saved as Digital Imaging and Communications in Medicine (DICOM) format. These images were imported Mimics20. 0 (Materialise Inc, Leuven, Belgium). After predefined threshold, region growing, mask modifying and preliminary borders smoothing, the project was outputted in STL format. This model was imported into Geomagic-Warp2017 (3DSystems Inc, Rock Hill, SC, USA) software for advanced smoothing, adjusting polygon mesh and fitting surface and also integrated into the Solidworks2017 (Dassault Systemes, Vélizy-Villacoublay, France).

**Fig. 1:**
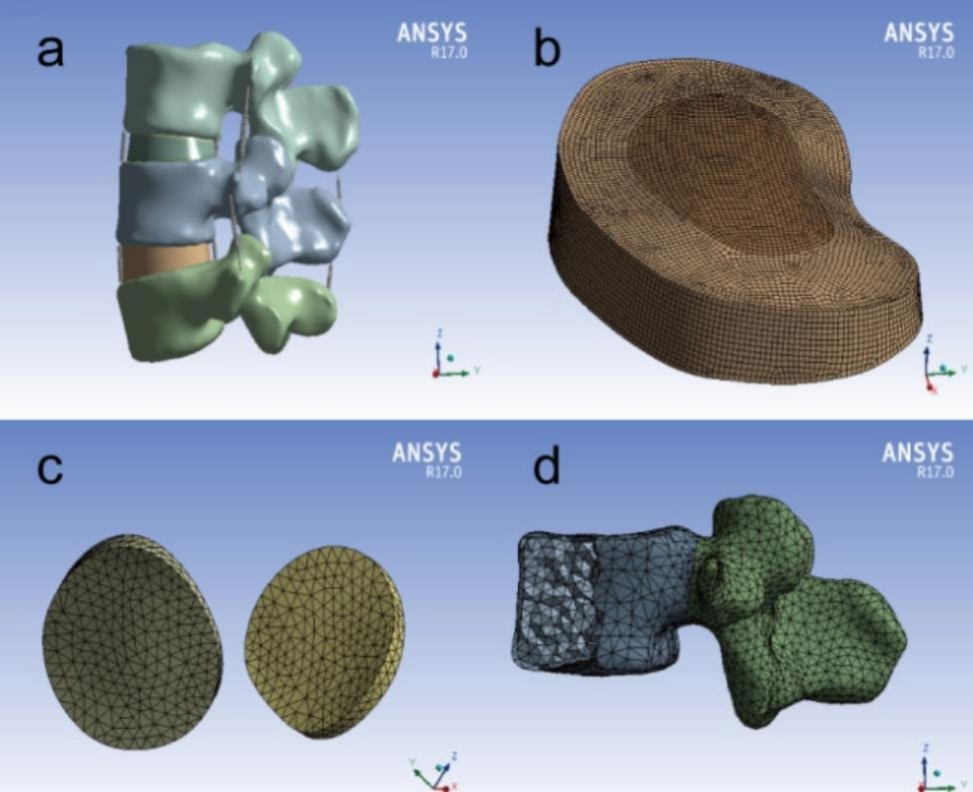
Details of models: (a) intact model, (b) intervertebral disc, (c) facet joint and articular cartilage, (d) uneven meshes of vertebral body

Cancellous bone and cortical bone were combined by Boolean logic operations;the thickness of cortical bone and bony endplates were set to 1 mm [23]. Facet joints and intervertebral discs were created in part pattern; articular cartilage filled the joint space with a thickness of 0. 2 mm to withstand rotational stress; the volume ratio of annulus fibrosus and nucleus pulposus was approximately 1:1 (Fig. 1).

### Development of SA LLIF model

**Fig. 2:**
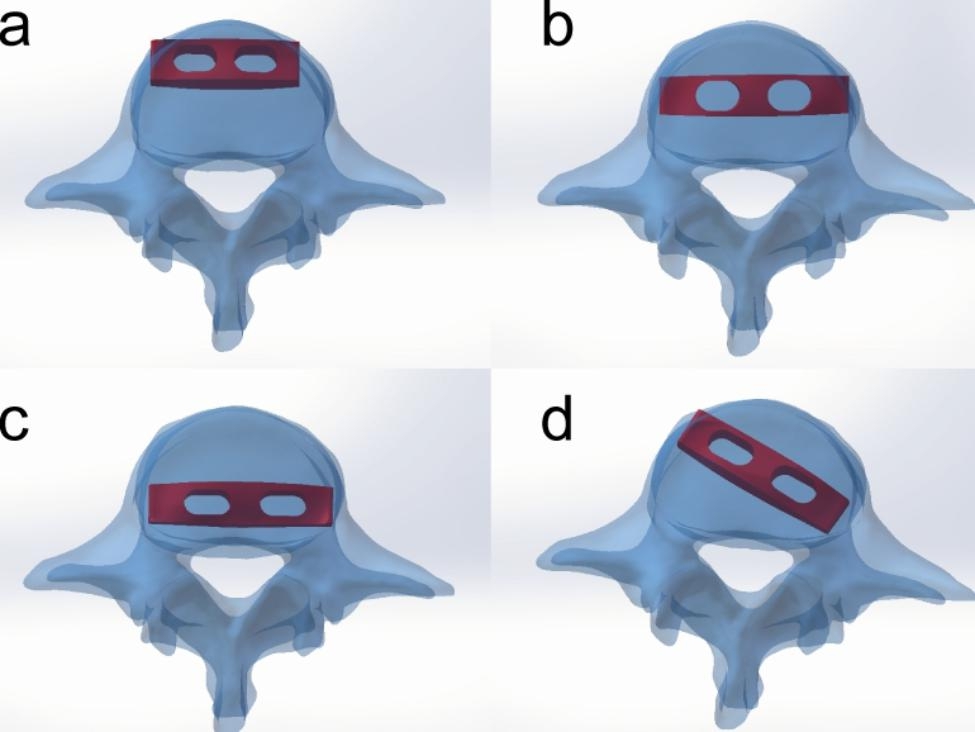
Different positions of cage: (a) anterior placement (AP), (b) middle placement(MP), (c) posterior placement (PP), (d) oblique placement (OP).

Due to a higher incidence of degeneration, L4-L5 segment, this segment was selected to simulate intervertebral fusion surgery. The model of interbody fusion cage was reconstructed with the prototype provided by Zimmer (8-10 mm height, 45 mm length, 17 mm width). The LLIF cage was placed into L4/5 disc space in four directions:anterior placement (AP), middle placement (MP), posterior placement (PP), oblique placement (OP) (Fig. 2). The cage of AP was close to the anterior edge of the endplate and parallel to the coronal midline. The cage of MP was close to the coronal midline of the endplate and perpendicular to the sagittal midline. The cage of PP was close to the posterior edge of the endplate and parallel to the coronal midline. The cage of OP was close to the centre of endplate and at 45 degree to the coronal midline. The cartilaginous endplate in L4/5 was removed, anterior longitudinal ligament and posterior structure were preserved in the meantime. The gap between the cage and the bony endplate was filled with cancellous bone. Some simplification of details were applicable in the modeling process, considering fault tolerance and reliability issues. In the end, all of the parts were detected by interference to evaluate their process quality, then were imported into Ansys 17. 0 (Ansys, Canonsburg, PA, USA).

### Parameter setting and meshing

We created a simplified model in which the various materials were recognized to be homogeneous. According to the previously set material property parameters [24, 25], the model including cortical bone, cancellous bone, cartilage endplates and interbody fusion cage were assigned to different elastic modulus and Poisson’s ratio. The details are presented in Table 1. The start and end points of the ligament were set according to anatomical characteristics, and their material properties were represented using cable elements such as spring patterns [26]. To improve the calculation accuracy, mixed mesh method was adopted to build meshes of the lumbar model, and different mesh types were established. This method allowed for more nodes and elements, which resulted in higher accuracy of biomechanical analysis (Fig. 1). The minimum effective size of four-node tetrahedron body element was 1.0 mm, which was used for facet joints.

**Table 1:** Mechanical parameters of spinal models

Material	Young’s modulus (MPa)	Poisson’s ratio	Cross-sectional area (mm^2^)
Cancellous bone	100	0.2	-
Cortical bone	12,000	0.3	-
Posterior bone	3500	0.25	-
Articular cartilages	35	0.4	-
Endplate	25	0.25	-
Annulus fibrosus	4.2	0.45	-
Nucleus pulposus	1	0.499	-
Spinal cage	3600	0.25	-
Capsular ligament	7.5	0.3	-
posterior longitudinal ligament	10 (< 11%) 20 (> 11%)	-	20
Ligament flavum	15 (< 6.2%) 19 (> 6.2%)	-	40
Interspinous ligament	10 (< 14%) 12 (> 14%)	-	40
Superspinous ligament	8 (< 20%) 15 (> 20%)	-	30
Intertansverse ligament	10 (< 18%) 59 (> 18%)	-	1.8
Anterior longitudinal ligament	7.8 (< 12%) 20 (> 12%)	-	63.7

### Boundary conditions and loads

L5 bottom was set as a fixed support surface, and six degrees of freedom were constrained. A vertical load of 400 N and the bending moment of 10 Nm were applied to the upper surface of the L3 vertebra to simulate movements of flexion, extension, lateral bending, and axial rotation. Contact types were defined as ‘Bonded’, except for the contact type between facet joint and cartilage, which was defined as ‘Frictionless’ [27].

### Evaluation criteria

Firstly, the range of motion (ROM) of all functional spinal units (FSU) were recorded, including surgical segment and adjacent segment. Secondly, the equivalent(von Mises)stress of endplate, facet joint and intervertebral fusion cage were observed.

## Result

### Model validity and mesh convergence verification

**Fig. 3:**
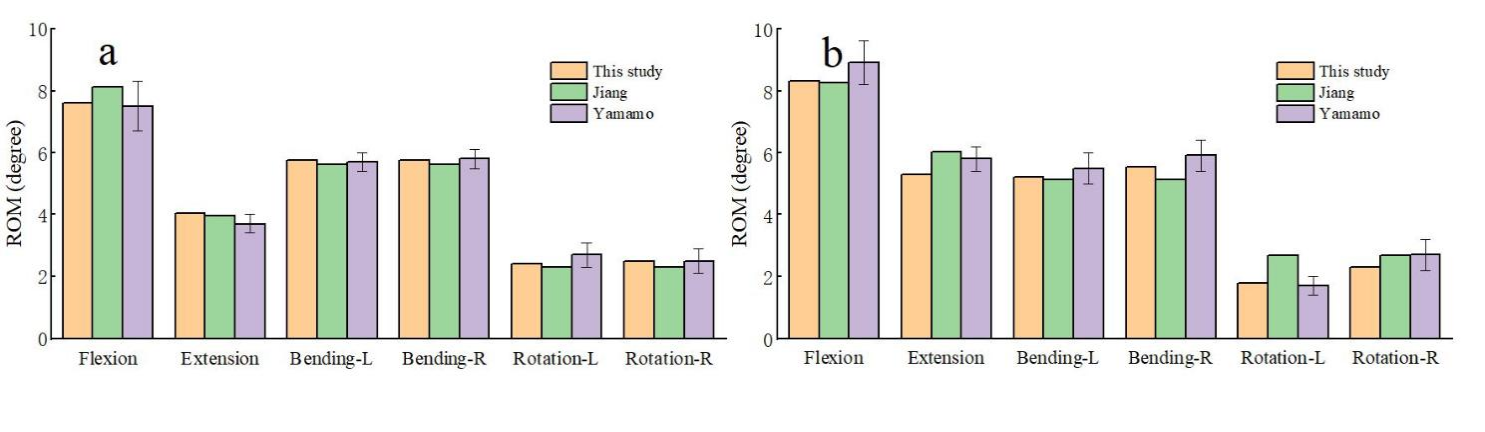
Comparison of ROM between the current intact finite element model and previous studies:(a) L3-4, (b) L4-5; L, left;R, right

**Fig. 4:**
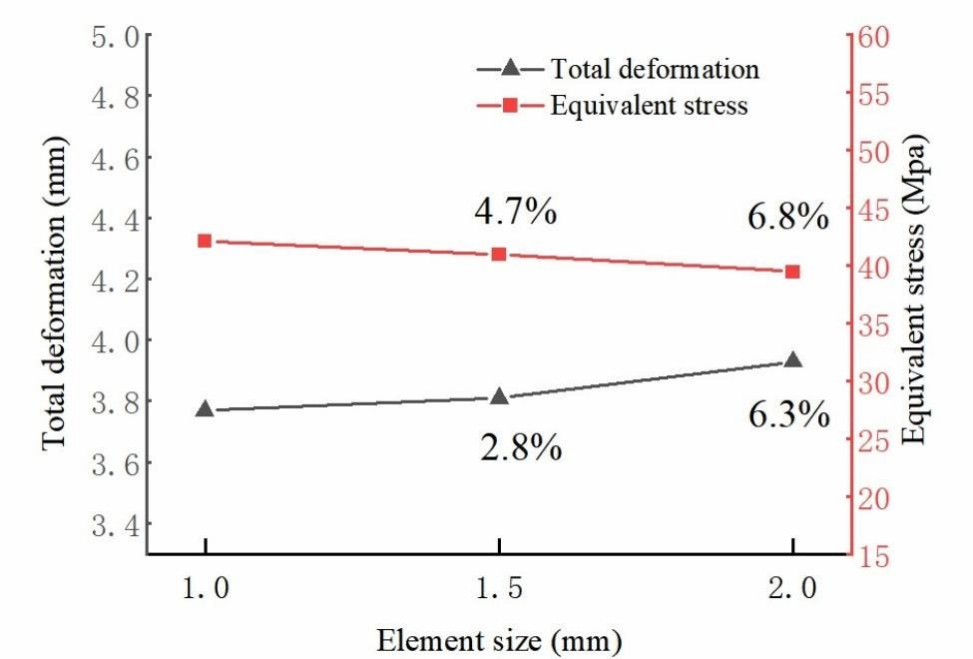
Result of meshing sensitivity study

Figure 3 indicates the result of model verification. The convergence of the model was verified by analyzing the maximum deformation and von-Mises stress in three element sizes (1 mm, 1.5 mm, 2 mm) models. The resolution difference between the predicted results of the 1.5 mm mesh size and 1 mm mesh size was < 5%, the 1.5 mm mesh was considered convergent (Fig. 4). Owing to modeling inconsistencies, some individual values were inconsistent with previous finite element results, however, all values in different conditions were within the vitro range (Yamamoto [28]).

### ROM of L4-5 and L3-4

**Fig. 5:**
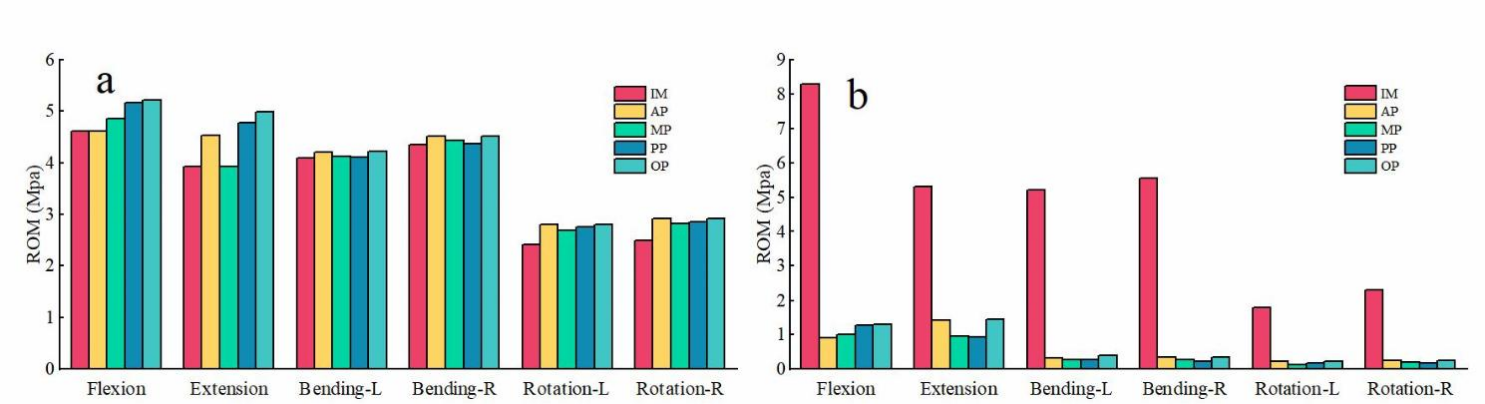
Changes in ROM before and after surgery: (a) L3-4, (b) L4-5; intact model (IM).

Figure 5 shows ROM of surgical and adjacent segment under various engineering conditions. When LLIF cage was used, a dramatic decline loss of motion at surgical level L4-5 occurred regardless the placement direction of intervertebral cage. The ROM of L4-5 was reduced by 84.21–89.03% in flexion, 72.64–82.26% in extension, 92.5-95.85% in right and left lateral bending, and 87.22–92.77% in right and left axial rotation, respectively. Meanwhile, ROM of L3-4 after LLIF was mildly increased by an average of 9.6% in all motion directions.

### Equivalent (von Mises) stress of endplate and cage

**Fig. 6:**
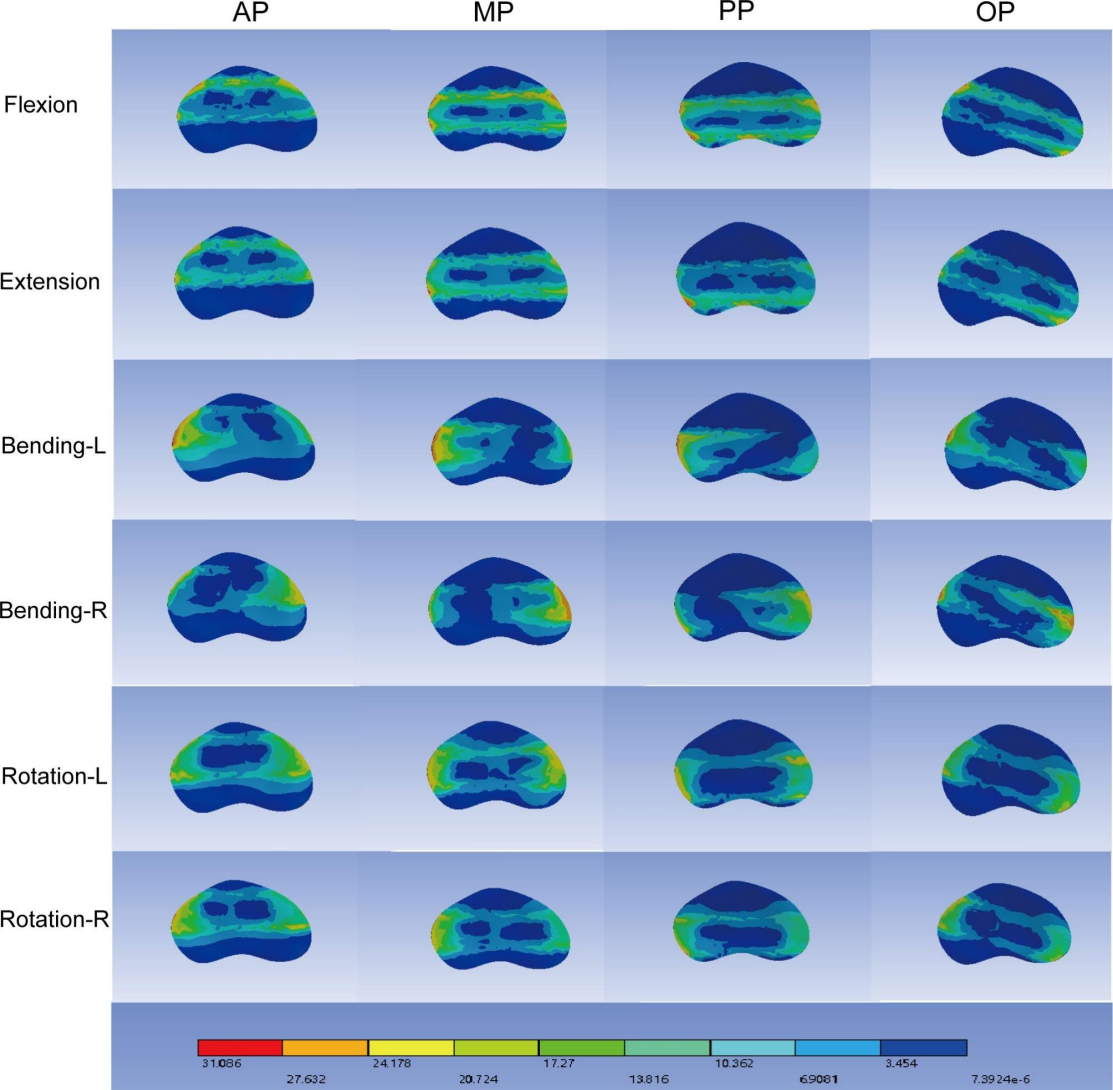
Stress nephogram of the L5 superior endplate

**Fig. 7:**
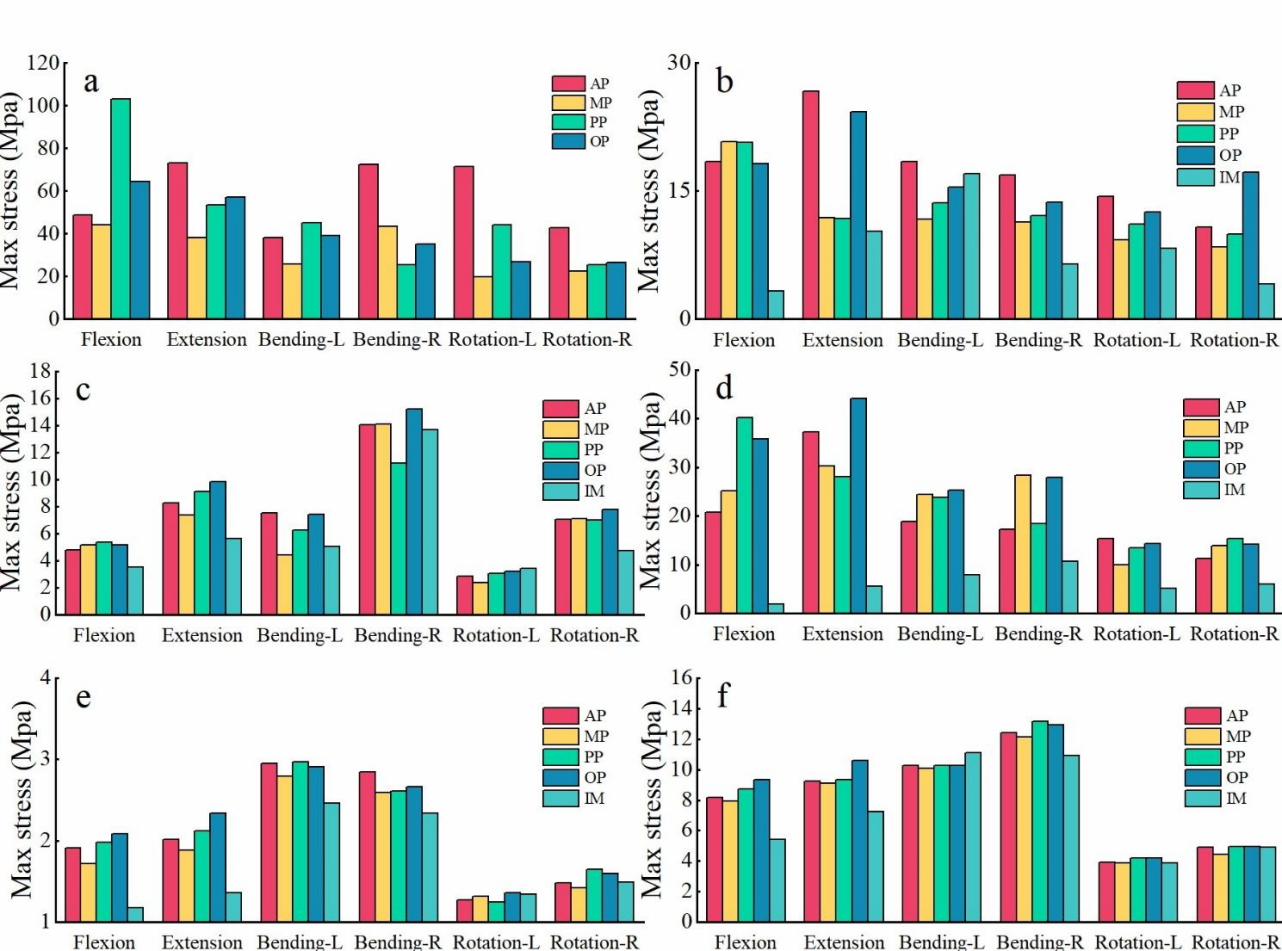
Details of maximum stress:(a) cage, (b) L5 superior endplate, (c) L4 superior endplate, (d) L4 inferior endplate, (e) Intervertebral disc, (f) facet joints

Interbody fusion cage subsidence and displacement often occur in the upper endplate postoperatively. Figure 6 shows the stress nephogram of the L5 superior endplate. The distribution of stress was consistent with the contact surface between cage and endplate. Figure 7(a-d) represents the maximum stress variation of cage and endplate. MP had the smallest stress peaks in most motion modes. The cage stress of MP was 43.95Mpa in flexion, 38.05Mpa in extension, 25.68Mpa in left bending, 43.53Mpa in right bending, 19.74Mpa in left rotation, and 22.44Mpa in right rotation.

### Stress of facet joints and proximal intervertebral disc

The maximum stress of facet joints and intervertebral disc are displayed in Fig. 7(e, f). Almost all stress peaks were increased after SA LLIF, including adjacent disc, facet joints, and endplates. As shown in our result, MP had the slightest tendency to raise the stress. The facet joints stress of MP was 7.93Mpa in flexion, 9.11Mpa in extension, 10.09Mpa in left bending, 12.13Mpa in right bending, 3.87Mpa in left rotation, and 4.43Mpa in right rotation. The L3/4 intervertebral disc stress of MP was 1.72 Mpa in flexion, 1.88Mpa in extension, 2.79Mpa in left bending, 2.59Mpa in right bending, 1.31Mpa in left rotation, and 1. 42Mpa in right rotation.

## Discussion

Undeniably, SA LLIF has clear advantages in appropriate cases in terms of clinical outcome and complications. Regarding the location of the cage in lumbar fusion, investigators have also conducted clinical and mechanics studies. A previous study [29] showed a lower cage center was more liable to cause adjacent facet joint degeneration (AFD) following the transforaminal lumbar interbody fusion (TLIF). Yan Liang [30] advised the cage should be placed in traverse orientation in TLIF. Unlike TLIF, SA LLIF does not have an auxiliary internal fixation and its stability is more dependent on intervertebral fusion. The anteriorly placed cage can contribute to restoration of segmental angle, whereas the posteriorly located cage might maintain the indirect decompression effect [31]. Although there are many perspectives on the position of the cage, the optimal position is a combination of low stress and sufficient stiffness. Therefore, we are dedicated to optimizing the cage position in SA LLIF. The aim is to explore the effect of less on the adjacent segments while maintaining stability through mechanical experiments.

In our finite element research, intact model and four commonly used clinical placements were reconstructed. From a mechanical point of view, excessive division of angles is meaningless, and simplified models can make trends in complex variables easier to understand and intuitive. In terms of model verification, many researchers have compared model simulations with vitro experiments. However, as an indirect validation method, segments motion does not fully represent the in vivo situation. Due to complex coupled loading patterns in the model, more detailed sensitivity analysis is necessary. Both indirect validation and sensitivity analysis of the grid were applied in this experiment. Of particular note is the number of mesh. A large amount of calculations can easily lead to computational failure. In addition, attention should also be paid to the application of tetrahedral and hexahedral meshes. For simple geometry, it relies more on an 8-node hexahedral mesh. While for curved boundary models, such as the posterior part of the vertebral body, more nodes and elements are needed to obtain more accurate solutions, or a quadratic tetrahedron is used. Excessive use of hexahedra will make the meshing process too long, prone to errors or even failure. Meanwhile, the inhomogeneous meshing method was used in this study. Under the condition of convergence, mainly 2 mm meshes were used, and smaller meshes were allowed for local contact points, such as facet joints. Thus, the accuracy of the operation could be improved without affecting the overall operation time.

The priority to be analyzed is the effect of cage position on the stability of the surgical segment. The ROM of the FE model can directly reflect the stability of the postoperative model. Sufficient stability of the postoperative FSU is beneficial for intervertebral bone fusion. In our study, compared with the IM, the ROM of four placements was significantly reduced. The variation in the magnitude of reduction of all placements was not very obvious. The elasticity modulus of cage is much greater than that of the disc, which in turn leads to increased segmental stiffness of the FUS. In terms of movement patterns, the most influential is the posterior extension. The ROM of the L4-5 segment in AP, MP, PP, OP mode under extension was significantly reduced by 73.01%, 82.07%, 82. 22%, 72.64%, respectively. It means that SA LLIF can effectively reduce the activity of the surgical segment (> 70%) at the index level.

Our results also indicated that the cage location was informative for cage subsidence, which was associated with segmental instability. The mechanical manifestation is the excessive stress on the contact surface. The stress distribution cloud showed that the stress was concentrated at the contact surface of the cage and the endplate. In terms of the stress peak in cage, MP had the lowest stress in the other motions, except for OP in right bending. More peak stress occurred in AP, which not indicated its stability. It is commonly believed that the smaller the internal fixation stress, the more stable it is. However, values in all tests are below the yield stress of cage, so stability should be determined comprehensively [32]. In addition, the size of deformation can also reflect the stability of segments to a certain extent. Our research proved that the minimum deformation occurred in MP under flexion and extension. The deformation in flexion, extension, left bending, right bending was 0. 4356 mm, 0. 4351 mm, 0. 0614 mm, 0. 0667 mm, respectively. The average displacement of AP was the largest, which was similar to the stress distribution of cage.

Compared with intact model, the maximum stress on the superior and inferior endplates of the surgical segment also increased significantly, the upper endplate stress of L5 was higher than the lower endplate stress of L4. The maximum stress of MP under flexion and extension conditions was similar to that of OP, and the peak stress of MP under bending and rotation conditions was smaller. The oblique position leads to uneven force during the movement, and the stress is too concentrated, resulting in a large force on the endplate. The stiffness of the cage is much greater than that of the endplate, so the stress trends in the cage and the endplate are not the same. In the view of stress variation, L5 upper endplate stress had more influence on settlement. Overall, middle placement (MP) showed lower stress in the endplate and cage.

The previous study [33–36] has indicated biomechanical changes following lumbar fusion may cause degeneration in adjacent segments. The reason is mainly the increased activity and stress in the adjacent segment. Our research showed that the ROM of adjacent segment (L3-4) increased slightly for all postures following SA LLIF. The increased activity of L3-4 was compensatory because of the enhanced stiffness of L4-5. Due to the greater stiffness, its activity increased even more. As the ROM increases, the discs and facet joints are exposed to greater stress, accelerating the degeneration of the discs and facet joints. In the upright situation, 70–90% of the static load is borne by the vertebral body and 10–20% of the axial load is borne by the facet joints [37]. In the case of body flexion, the facet joint bears more shear stress. As shown in our results, under flexion and extension, the stress on the small joint increased after SA LLIF. In comparison, the stress increase in the facet joints of MP was less than in other positions. Similarly, the disc pressure in the adjacent segment also changed in the SA LLIF models. As shown in our results, the interbody disc stress of L3/4 had increased in flexion, extension and bending. MP had the smallest increment, increasing by 47% in flexion, 38%in extension, 14% in left lateral bending, 11% in right bending. Excessive loading can lead to mechanical failure of the intervertebral disc. Further, these loads may lead to the rupture of central zone in the end plate. Common structural degeneration includes reduced nucleus pulposus volume, loss of disc height, and tearing of the annulus fibrosus. Structural degeneration of the intervertebral disc is often accompanied by functional degeneration, which can cause clinical symptoms. In essence, the change of intervertebral disc force of the adjacent segment is also an indicator for judging the adjacent segment degeneration (ASD), consistent with the conclusions of Du [38]. Therefore, we believe that the MP has the least effect on the cephalad adjacent segment among all positions.

Our research was based on finite element analysis and has some inadequacies. First, modeling of geometry varied slightly due to individual differences in volunteers and differences in accuracy during CT scanning. Second, models were simplified to analyze the trend of force changes. For example, the effects of skeletal muscle and other soft tissues were ignored, and the variation in bone density in different regions of the endplate was not considered. Full spine modeling was not performed, which could lead to some deviations in the specific values, but would not affect the overall trend. Third, the effect of the front convex angle and size of the cage on the mechanical performance were neglected. Our next research will focus on topology optimization of the cage in SA LLIF.

## Conclusion

In our study, SA LLIF risked accelerating the adjacent segment degeneration. The cage position had an influence on the distribution of endplate stress and the magnitude of facet joint stress. Compared with other positions, MP had the slightest effect on the stress in the adjacent facet joints. Meanwhile, MP seems to play an important role in reducing the risk of cage subsidence.

## Data Availability

The datasets used and/or analysed during the current study are available from the corresponding author on reasonable request.

## Abbreviations

SA LLIF: stand-alone lateral lumbar interbody fusion
AP: anterior placement
MP: middle placement
PP: posterior placement
OP: oblique placement
ROM: range of motion
ALIF: anterior lumbar interbody fusion
TLIF: transforaminal lumbar interbody fusion
PLIF: posterior lumbar interbody fusion
ASD: adjacent segment disease
FEA: finite element analysis
DICOM: digital Imaging and Communications in Medicine
FSU: functional spinal units
VAS: Visual Analogue Scale
ODI: Oswestry Disability Index

## References

[1] Ozgur BM, Aryan HE, Pimenta L, Taylor WR (2006). Extreme Lateral Interbody Fusion (XLIF): a novel surgical technique for anterior lumbar interbody fusion. Spine J.

[2] Li J, Sun Y, Guo L, Zhang F, Ding W, Zhang W (2022). Efficacy and safety of a modified lateral lumbar interbody fusion in L4-5 lumbar degenerative diseases compared with traditional XLIF and OLIF: a retrospective cohort study of 156 cases. BMC Musculoskelet Disord.

[3] Brau SA (2002). Mini-open approach to the spine for anterior lumbar interbody fusion: description of the procedure, results and complications. Spine J.

[4] Epstein NE (2019). Review of Risks and Complications of Extreme Lateral Interbody Fusion (XLIF). Surg Neurol Int.

[5] Hah R, Kang HP (2019). Lateral and Oblique Lumbar Interbody Fusion-Current Concepts and a Review of Recent Literature. Curr Rev Musculoskelet Med.

[6] Kwon B, Kim DH (2016). Lateral Lumbar Interbody Fusion: Indications, Outcomes, and Complications. J Am Acad Orthop Surg.

[7] Taba HA, Williams SK (2020). Lateral Lumbar Interbody Fusion. Neurosurg Clin N Am.

[8] Screven R, Pressman E, Rao G, Freeman TB, Alikhani P (2021). The Safety and Efficacy of Stand-Alone Lateral Lumbar Interbody Fusion for Adjacent Segment Disease in a Cohort of 44 Patients. World Neurosurg.

[9] Metzger MF, Robinson ST, Maldonado RC, Rawlinson J, Liu J, Acosta FL (2017). Biomechanical analysis of lateral interbody fusion strategies for adjacent segment degeneration in the lumbar spine. Spine J.

[10] Louie PK, Varthi AG, Narain AS (2018). Stand-alone lateral lumbar interbody fusion for the treatment of symptomatic adjacent segment degeneration following previous lumbar fusion. Spine J.

[11] Chioffe M, McCarthy M, Swiatek PR (2019). Biomechanical Analysis of Stand-alone Lateral Lumbar Interbody Fusion for Lumbar Adjacent Segment Disease. Cureus.

[12] Cappuccino A, Cornwall GB, Turner AW (2010). Biomechanical analysis and review of lateral lumbar fusion constructs. Spine (Phila Pa 1976).

[13] Fang G, Lin Y, Wu J (2020). Biomechanical Comparison of Stand-Alone and Bilateral Pedicle Screw Fixation for Oblique Lumbar Interbody Fusion Surgery-A Finite Element Analysis. World Neurosurg.

[14] Ahmadian A, Bach K, Bolinger B (2015). Stand-alone minimally invasive lateral lumbar interbody fusion: multicenter clinical outcomes. J Clin Neurosci.

[15] Marchi L, Abdala N, Oliveira L, Amaral R, Coutinho E, Pimenta L (2013). Radiographic and clinical evaluation of cage subsidence after stand-alone lateral interbody fusion. J Neurosurg Spine.

[16] Yang H, Liu J, Hai Y (2021). Is instrumented lateral lumbar interbody fusion superior to stand-alone lateral lumbar interbody fusion for the treatment of lumbar degenerative disease? A meta-analysis. J Clin Neurosci.

[17] Abbushi A, Cabraja M, Thomale UW, Woiciechowsky C, Kroppenstedt SN (2009). The influence of cage positioning and cage type on cage migration and fusion rates in patients with monosegmental posterior lumbar interbody fusion and posterior fixation. Eur Spine J.

[18] Yuan W, Kaliya-Perumal AK, Chou SM, Oh JY. Does Lumbar Interbody Cage Size Influence Subsidence? A Biomechanical Study. Spine (Phila Pa 1976). 2020. 45(2): 88–95. 10.1097/BRS.0000000000003194 31415458

[19] Alimi M, Lang G, Navarro-Ramirez R (2018). The Impact of Cage Dimensions, Positioning, and Side of Approach in Extreme Lateral Interbody Fusion. Clin Spine Surg.

[20] Jin J, Ryu KS, Hur JW, Seong JH, Kim JS, Cho HJ (2018). Comparative Study of the Difference of Perioperative Complication and Radiologic Results: MIS-DLIF (Minimally Invasive Direct Lateral Lumbar Interbody Fusion) Versus MIS-OLIF (Minimally Invasive Oblique Lateral Lumbar Interbody Fusion). Clin Spine Surg.

[21] Qiao G, Feng M, Liu J, et al. Does the Position of Cage Affect the Clinical Outcome of Lateral Interbody Fusion in Lumbar Spinal Stenosis. Global Spine J. 2020: 2192568220948029. 10.1177/2192568220948029 PMC8907639 32856471

[22] Hiyama A, Katoh H, Sakai D, Sato M, Tanaka M, Watanabe M (2021). Cluster analysis to predict factors associated with sufficient indirect decompression immediately after single-level lateral lumbar interbody fusion. J Clin Neurosci.

[23] Lu T, Lu Y (2019). Comparison of Biomechanical Performance Among Posterolateral Fusion and Transforaminal, Extreme, and Oblique Lumbar Interbody Fusion: A Finite Element Analysis. World Neurosurg.

[24] Ahn YH, Chen WM, Lee KY, Park KW, Lee SJ (2008). Comparison of the load-sharing characteristics between pedicle-based dynamic and rigid rod devices. Biomed Mater.

[25] Guo HZ, Zhang SC, Guo DQ (2020). Influence of cement-augmented pedicle screws with different volumes of polymethylmethacrylate in osteoporotic lumbar vertebrae over the adjacent segments: a 3D finite element analysis. BMC Musculoskelet Disord.

[26] Jones AC, Wilcox RK (2008). Finite element analysis of the spine: towards a framework of verification, validation and sensitivity analysis. Med Eng Phys.

[27] Wang L, Kang J, Shi L (2018). Investigation into factors affecting the mechanical behaviours of a patient-specific vertebral body replacement. Proc Inst Mech Eng H.

[28] Yamamoto I, Panjabi MM, Crisco T, Oxland T (1989). Three-dimensional movements of the whole lumbar spine and lumbosacral joint. Spine (Phila Pa 1976).

[29] Li F, Zhan X, Xi X (2021). Do the positioning variables of the cage contribute to adjacent facet joint degeneration? Radiological and clinical analysis following intervertebral fusion. Ann Transl Med.

[30] Liang Y, Zhao Y, Xu S, Zhu Z, Liu H, Mao K (2020). Effects of Different Orientations of Cage Implantation on Lumbar Interbody Fusion. World Neurosurg.

[31] Park SJ, Lee CS, Chung SS, Kang SS, Park HJ, Kim SH (2017). The Ideal Cage Position for Achieving Both Indirect Neural Decompression and Segmental Angle Restoration in Lateral Lumbar Interbody Fusion (LLIF). Clin Spine Surg.

[32] Biswas JK, Rana M, Majumder S, Karmakar SK, Roychowdhury A (2018). Effect of two-level pedicle-screw fixation with different rod materials on lumbar spine: A finite element study. J Orthop Sci.

[33] Lee JC, Choi SW (2015). Adjacent Segment Pathology after Lumbar Spinal Fusion. Asian Spine J.

[34] Li XH, She LJ, Zhang W, Cheng XD, Fan JP (2022). Biomechanics of extreme lateral interbody fusion with different internal fixation methods: a finite element analysis. BMC Musculoskelet Disord.

[35] Chen CS, Cheng CK, Liu CL, Lo WH. Stress analysis of the disc adjacent to interbody fusion in lumbar spine. Med Eng Phys. 2001;23(7):483 – 91. 10.1016/s1350-4533(01)00076-5. 36. Guigui P, Wodecki P, Bizot P, Lambert P, Chaumeil G, Deburge A. [Long-term influence of associated arthrodesis on adjacent segments in the treatment of lumbar stenosis: a series of 127 cases with 9-year follow-up]. Rev Chir Orthop Reparatrice Appar Mot. 2000;86(6):546 – 57. 11060428

[37] Adams MA, Hutton WC (1980). The effect of posture on the role of the apophysial joints in resisting intervertebral compressive forces. J Bone Joint Surg Br.

[38] Du CF, Cai XY, Gui W (2021). Does oblique lumbar interbody fusion promote adjacent degeneration in degenerative disc disease: A finite element analysis. Comput Biol Med.

